# Meta-Analysis of Cancer Triploidy: Rearrangements of Genome Complements in Male Human Tumors Are Characterized by XXY Karyotypes

**DOI:** 10.3390/genes10080613

**Published:** 2019-08-13

**Authors:** Ninel M. Vainshelbaum, Pawel Zayakin, Regina Kleina, Alessandro Giuliani, Jekaterina Erenpreisa

**Affiliations:** 1 Cancer Research Division, Latvian Biomedical Research and Study Centre, LV-1067 Riga, Latvia; 2 Faculty of Biology, The University of Latvia, LV-1586 Riga, Latvia; 3 Department of Pathology, Riga Stradins University, LV-1007 Riga, Latvia; 4 Environment and Health Department, Istituto Superiore di Sanità, 00161 Rome, Italy

**Keywords:** cancer near-triploidy, male tumors, karyotype meta-analysis, XXY, whole-genome rearrangements, digyny

## Abstract

Triploidy in cancer is associated with poor prognosis, but its origins remain unclear. Here, we attempted to differentiate between random chromosomal and whole-genome origins of cancer triploidy. *In silico* meta-analysis was performed on 15 male malignant and five benign tumor cohorts (2928 karyotypes) extracted from the Mitelman Database, comparing their ploidy and combinations of sex chromosomes. A distinct near-triploid fraction was observed in all malignant tumor types, and was especially high in seminoma. For all tumor types, X-chromosome doubling, predominantly observed as XXY, correlated strongly with the near-triploid state (*r* ≈ 0.9, *p* < 0.001), negatively correlated with near-diploidy, and did not correlate with near-tetraploidy. A smaller near-triploid component with a doubled X-chromosome was also present in three of the five benign tumor types, especially notable in colon adenoma. Principal component analysis revealed a non-random correlation structure shaping the X-chromosome disomy distribution across all tumor types. We suggest that doubling of the maternal genome followed by pedogamic fusion with a paternal genome (a possible mimic of the fertilization aberration, 69, XXY digyny) associated with meiotic reprogramming may be responsible for the observed rearrangements of genome complements leading to cancer triploidy. The relatively frequent loss of the Y-chromosome results as a secondary factor from chromosome instability.

## 1. Introduction

Aneuploidy (an abnormal number of chromosomes) is a well-known hallmark of malignant tumors and is generally associated with their aggressive development [1,2]. With results of cancer genome sequencing projects revealing flaws in the mutation theory, its inability to explain chemoresistance, and for providing targeted therapies in general with limited clinical benefit, the aneuploidy theory of cancer proposed in the 19th century by David Hansemann and Theodor Boveri is enjoying a renaissance [3]. The aneuploidy theory posits that genome instability is causally responsible for the propagation of cancer. Currently, the evolution of cancer is viewed as a system behavior of a stress response with adaptive advantages of microevolution (including the Darwinian selection of the fittest mutant clones), which is followed by the further destabilization of the genome, leading to its crisis (chromothripsis), which results in a rapid and massive genome reorganization (punctuated evolution) unifying the diverse chromosomal and nuclear abnormalities [4,5]. The earlier stages of carcinogenesis in this setting still remain obscure [6,7]. Recent advances in the molecular characterization of aneuploidy revealed that the search for the general mechanism of how aneuploidy contributes to cancer is becoming increasingly challenging: It appears that aneuploidy can be linked to diverse molecular pathways [8] and favors both tumor-suppressing and driving effects [9,10]. Another problem in the advance of chaotic adaptations in cancer evolution; this is the so-called “Muller’s ratchet” (Muller 1964), which was proved experimentally on bacteria [11]. In his works, H. Muller postulated that neutral and harmful mutations (also inevitably caused by structural chromosome imbalances) should sooner or later lead to the extinction of asexual species. However, such unicellular species have existed on Earth for aeons. The same relates to somatic tumors of mammals; as with protists, they are immortal and resistant to extinction, and we still do not have an answer for why this is the case. Interestingly, some agamic protists return in a cycle from heterogenic aneupolyploidy in the interphase and chromatin diminution in the endoprophase to strict euploidy in each metaphase and telophase [12,13], likely compensating for Muller’s ratchet by gene conversion in the polyploidy phase [14]. Moreover, chromothripsis, which affects the chromosome order, may cause the de-speciation of mammalian tumor cells. Indeed, the atavistic recapitulation in human tumor cells of the unicellular programs is becoming apparent [15,16,17]. Interestingly, this epigenetic shift is also associated with polyploidy [18,19,20], whereas polyploidy and aneuploidy in human cancer cells are linked, in turn, with chromothripsis [21], genome reprogramming, and the upregulation of a single cell organism gene module [3,18,20,22]. Thus, cancer aneuploidy involves both genetic and crucial epigenetic changes, requiring deliberate escape from the mitotic control.

It therefore appears that an exit from the “blind corner” of Muller’s ratchet may be provided by the option for the aneupolyploid genome to convert chaos into order using the ploidy cycle as it operates with the genome complements, in contrast to the mitotic cycles, whose task is to orderly segregate chromosomes. The reproductive ploidy (life) cycles are doubling and halving the whole genome complements in order with the help of meiosis, performing recombination and reduction [23,24]. The elements of this meiosis-like mechanism are likely implemented by the reprogrammed cancer cells in the asexual or parasexual “life-cycles”, which are reciprocally joined in tumors with mitotic cycles [25,26,27,28].

Among aneuploidies of solid tumors, a near-triploid karyotype is often a hallmark of chemotherapy resistance and thus of increased survival potential [29,30]. We paid attention to the fact that among the numerical sex chromosome aberrations in the tumors of male patients, the assertive acquisition of an extra X-chromosome and frequent loss of the Y-chromosome have been reported in several cases [31,32,33,34,35,36,37], particularly in association with triploidy in the male germ cell tumor seminoma [38].

Here, we decided to use the advantage of the presence of two different sex chromosomes, X and Y, in a normal diploid male karyotype, in an attempt to differentiate between two potential constituents of tumor near-triploidy: The chromosomal aberrations and the rearrangements of the whole-genome complements. For this purpose, we performed an in silico meta-analysis of the male tumor karyotypes deposited in the Mitelman Database of Chromosome Aberrations and Gene Fusions in Cancer [39].

## 2. Materials and Methods

The karyotypes from 15 male malignant solid tumor types (untreated and presented in the >50 number of cases), epithelial and mesenchymal, somatic and germinative, and karyotypes from five benign tumor types were obtained from the Mitelman Database of Chromosome Aberrations and Gene Fusions in Cancer [39] in the period of January–March, 2019. None of the male patient karyotypes were affected with congenital sex chromosome aberrations such as Kleinfelter syndrome. The types of tumors and the number of patient karyotypes for each of them are presented in Table 1.

The tumor nomenclature used was based on the International Classification of Diseases for Oncology (ICD-O), the Systematized Nomenclature of Medicine (SNOMED), and the WHO Classification of Tumours of Soft Tissue and Bone—the same sources as the Mitelman database’s nomenclature. Seminoma was the germ cell tumor. Among somatic tumors, the lung carcinoma cohort included a total of five lung tumor types (squamous cell carcinoma, adenosquamous carcinoma, adenocarcinoma, undifferentiated large cell carcinoma, and small cell carcinoma), united from the evidence that both bronchoepithelial and neuroendocrine lung stem cells likely have one common precursor [40]. The gastric carcinoma cohort comprised adenocarcinoma and undifferentiated carcinoma. These cases were not sorted by stages of the malignant process in the Mitelman database. Only monoclonal karyotypes comprising 2928 tumor cases in total were collected, filtering out the cases with polyclonal karyotypes, cases where several samples were obtained from one patient, and incomplete sex chromosome karyotypes. Using the data analysis tools of the numpy [41], pandas [42], and scipy [43] Python libraries, a statistical analysis of the available data was performed to determine the relationship between modal chromosome numbers and different sex chromosome karyotypes.

The 2013 edition of the International System for Human Cytogenetic Nomenclature (ISCN) defines near-triploidy as a modal chromosome number that falls in the 58–80 range [44]. In our study, the boundaries of triploidy were also narrowed to a medium-sized range spanning 62–76 chromosomes, and a narrower range spanning 66–72 chromosomes. The nomenclature of the sex chromosome karyotypes was used as presented in the Mitelman database using ISCN, where a sex chromosome complement is expressed as related to ploidy level.

Principal component analysis (PCA) was applied to the X-chromosome disomy (#X-disomy) distribution across different tumor types in order to check for the departure of the #X-disomy patterns from randomness [45]. The departure from randomness of such a solution was estimated by means of the Bartlett-corrected chi-square as applied to maximum-likelihood factor extraction [46].

## 3. Results

### 3.1. Analysis of the Histograms of the Modal Chromosome Numbers in 15 Cohorts of Malignant Tumors

**Figure 1:**
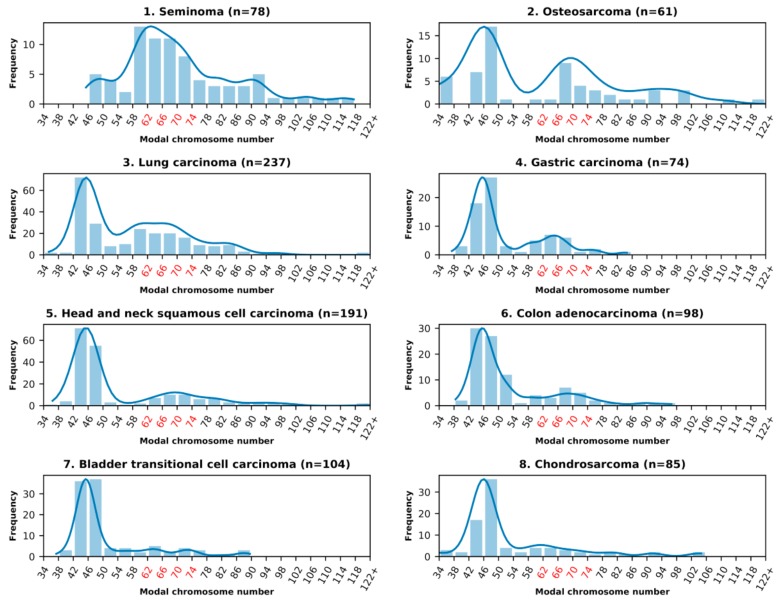

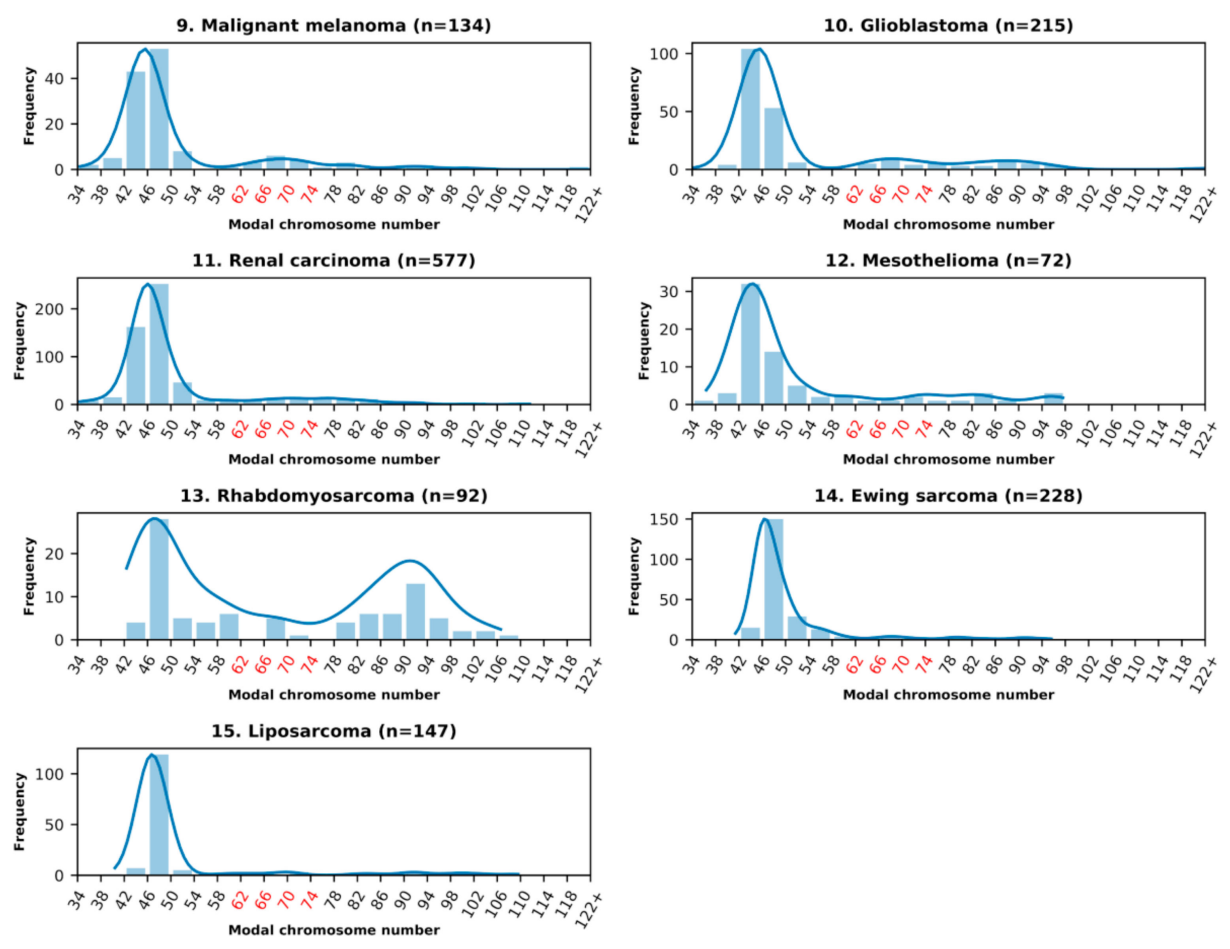
The modal chromosome number frequency histograms of 15 malignant tumor cohorts, numbered as listed in Table 1. The chromosome numbers within the (arbitrarily chosen) range of near-triploidy (62–76 chromosomes) are marked red.

In all examined malignant solid tumor types, listed in Table 1, the aneuploid karyotypes were present. The summary histograms of the modal chromosome numbers of each cohort are presented in Figure 1. 

It is seen that they include near-diploid karyotypes, near-triploid karyotypes, a degree of tetraploidy (high in rhabdomyosarcoma), and in some cases also hyper-tetraploid karyotypes. The near-triploid karyotypes were present in all malignant tumor types. Their percentage share for malignant tumor types 1–15 is presented in Table 1 in descending order. In particular, a high proportion of near-triploidy (42%) was observed for the germ tumor seminoma. In 14 examined somatic malignant tumor types, both epithelial and mesenchymal, the near-diploid karyotypes were predominating, while the proportion of near-triploid ones was less pronounced than in seminoma, albeit in a varying degree (Table 1, Figure 1). Osteosarcoma was a leader in triploidy (28%). Lung carcinoma also displayed a high proportion of near-triploid karyotypes (27%); other somatic tumors showed lower values.

### 3.2. Analysis of the Sex Chromosome Sets with #X-Disomy in Each Malignant Tumor Cohort in Relation to Ploidy for their Karyotypes

We also analyzed the sex chromosome sets with #X-disomy, which are presented alongside their percentage for each malignant tumor cohort in Table 1. It can be seen that the configuration XXY dominates in seminoma and is also predominant among #X-disomic karyotypes in 10 of 14 somatic malignant tumors. However, the proportion of the XX,-Y set is larger in them than in seminoma, while in head and neck (HN) squamous cell carcinoma, XX,-Y prevails over XXY. Other karyotypes with #X-disomy (XY,+X) + (X,-Y,+X) were a minority, with the exception of colon adenocarcinoma, where their proportion was comparatively high. Some of the (largely near-triploid) XXY karyotypes were also shown to possess an extra Y chromosome (especially evident in seminoma, osteosarcoma and colon adenocarcinoma); their percentage share is presented in Table 1.

**Figure 2:**
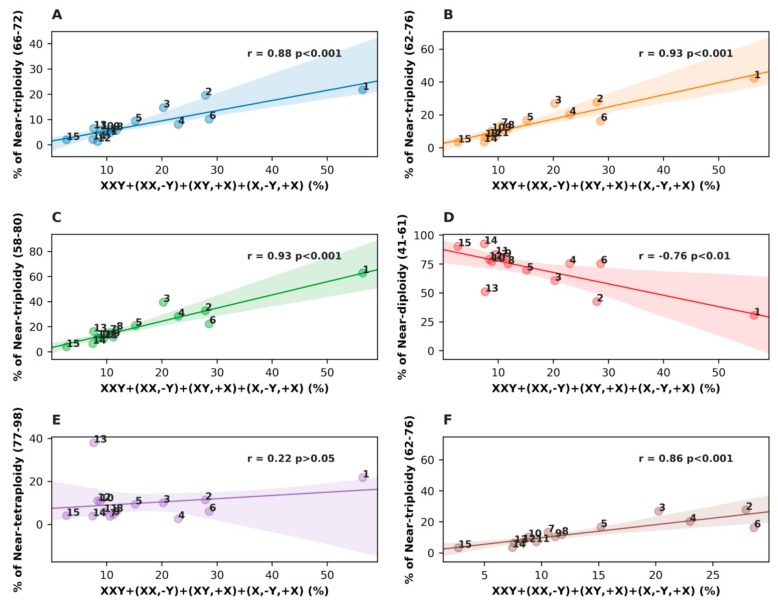
Results of the Pearson correlation analysis for all 15 patient karyotype cohorts of malignant tumors, evaluating the relationship between all karyotypes containing doubled X-chromosomes and ploidy in different chromosome ranges: (**A**) In relation to the narrow triploidy range (66–72 chromosomes); (**B**) in relation to the median triploidy range (62–76 chromosomes); (**C**) in relation to the wide (International System for Human Cytogenetic Nomenclature, ISCN) triploidy range (58–80 chromosomes); (**D**) in relation to the near-diploidy range (41–61 chromosomes); (**E**) in relation to near-tetraploidy range (77–98 chromosomes); and (**F**) only malignant somatic tumors are presented as related to the near-triploidy median range. The tumor cohort numbers are the same as in Table 1.

Further, we compared the relationship of the karyotypes exhibiting #X-disomy with different ploidy ranges of the modal chromosome numbers for all malignant tumors and for only somatic tumor cohorts, excluding seminoma. The results of this comparative statistical analysis are shown in Figure 2.

Strikingly, in spite of the many-fold differences in the proportions of the near-triploid karyotypes among 15 malignant tumor types, taken together, they provided a very high Pearson correlation between #X-disomic sex chromosome karyotypes and all tested ranges of near-triploidy, from the narrower range (*r* = 0.88; *p* < 0.001) to the median and widest range, equally (*r* = 0.93, *p* < 0.001) (Figure 2A–C). In the near-diploidy range (Figure 2D), the correlation was strongly negative (*r* = −0.76, *p* < 0.01), while in the near-tetraploidy range (Figure 2E), no correlation was observed. When excluding seminoma, the Pearson correlation of the somatic malignant tumor cohorts presented in Figure 2F in the median near-triploidy range was also convincingly strong (*r* = 0.86, *p* < 0.001).

**Figure 3:**
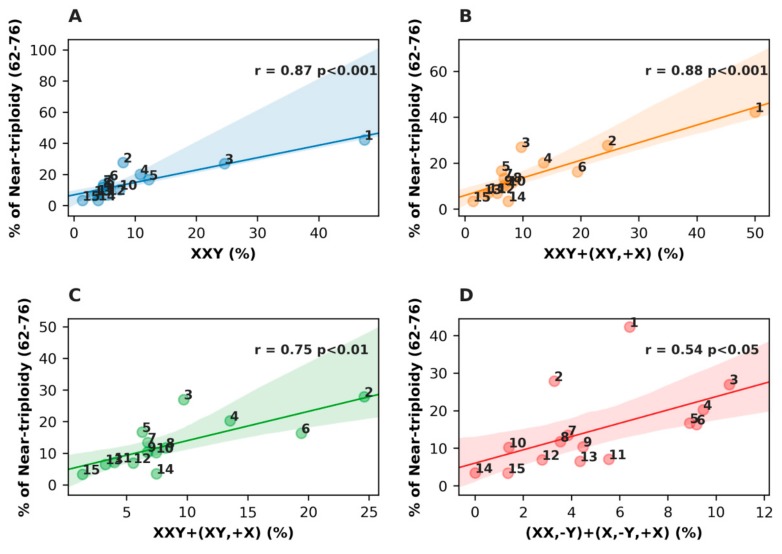
Results of the Pearson correlation analysis for malignant tumors evaluating the relationship between different karyotypes containing disomic X-chromosomes and ploidy in the median near-triploidy range. (**A**) For XXY and (**B**) XXY+(XY,+X) configurations; (**C**) X-disomic karyotypes with a Y chromosome; and (**D**) X-disomic karyotypes lacking a Y chromosome. The tumor cohort numbers are the same as in Table 1.

Further, we examined the influence of different #X-disomic karyotypes on Pearson correlation in the median range of near-triploidy (62–78 chromosomes). The results are presented in Figure 3. 

The results show a very high contribution of XXY karyotypes (Figure 3A) in the near-triploidy range (*r* = 0.88, *p* < 0.001), which remains the same, adding also near-diploid karyotypes with #X-disomy (Figure 3B). For somatic malignant tumors only, as presented in Figure 3C, this correlation with both karyotypes is smaller but still strong (*r* = 0.75, *p* < 0.01). The loss of #Y from #X-disomic near-triploid and near-diploid karyotypes presented for all malignant tumors in Figure 3D weakened the correlation with near-triploidy, but it still remained positive and statistically significant (*r* = 0.54, *p* < 0.05).

### 3.3. Analysis of All Sex Chromosome Configurations in Relation to Near-Triploidy in Malignant Tumors

**Figure 4:**
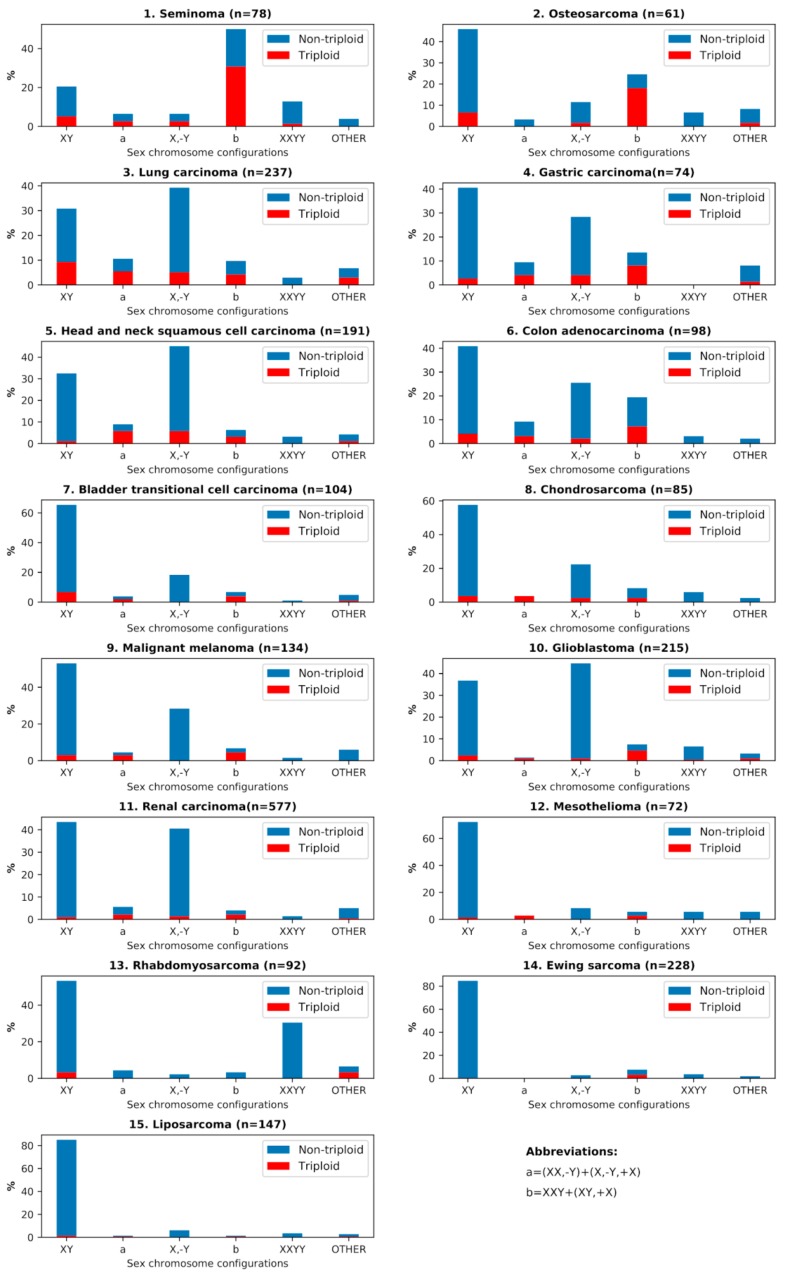
The percentages of different sex chromosome configurations and their respective percentages of near-triploidy (62–76 chromosomes) for all malignant tumor cohorts, numbered as listed in Table 1.

The relationship between near-triploidy (62–76) and all sex chromosome sets is presented in a bar-plot form for all malignant tumors in Figure 4. Besides the already discussed issues of the prevailing association of #X-disomy with near-triploidy, Figure 4 also reveals that a small portion of XY karyotypes and X,-Y karyotypes are also near-triploid; in particular, this is pronounced in lung carcinoma. This is likely to be associated with the chromosome instability processes. Contrary to the karyotypes with a doubled X-chromosome, the compositions of sex chromosomes with doubled Y and one or an absent X (XYY or YY) were rare (and therefore not presented in this article), while 10 of 16 tumor types (seminoma, osteosarcoma, lung carcinoma, colon adenocarcinoma, gastric carcinoma, bladder transitional cell carcinoma, liposarcoma, chondrosarcoma, Ewing sarcoma, and glioblastoma) were lacking them. Only one near-triploid XYY karyotype was found in the entire analyzed dataset, in rhabdomyosarcoma.

**Figure 5:**
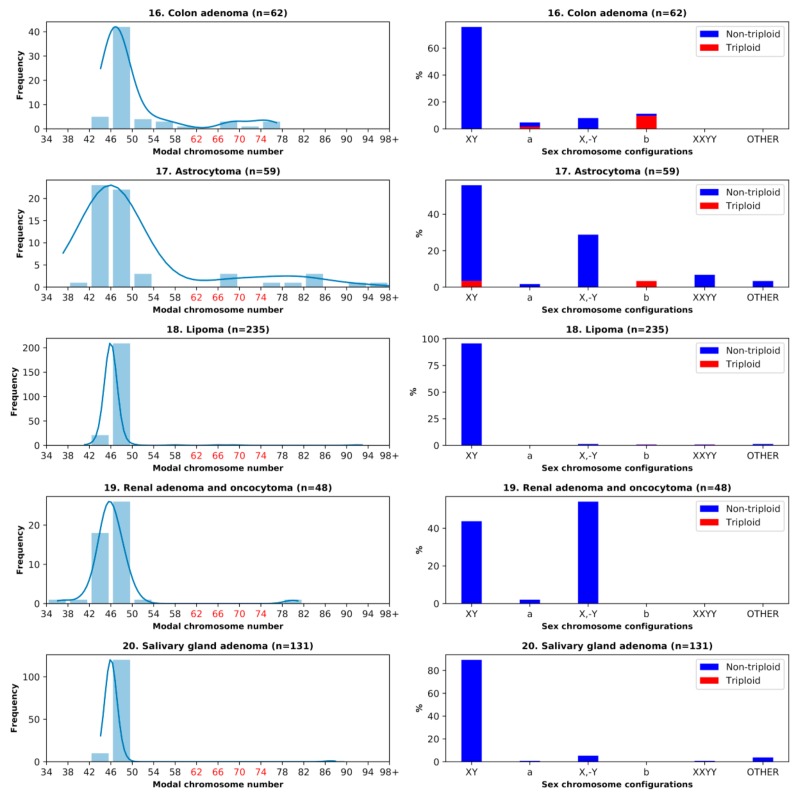
Benign tumor karyotypes. Left column: The histograms of the modal chromosome numbers, with near-triploidy marked red. Right column: The corresponding percentages of different sex chromosome configurations with #X-disomy and their respective percentages of near-triploidy (62–76 chromosomes) for five benign tumor cohorts. Designations (a,b) in the right column: (a) #X-disomic karyotypes lacking a Y chromosome (XX,-Y and X,-Y,+X); (b) #X-disomic karyotypes with a Y chromosome (XXY and XY,+X).

As triploidy in association with #X-disomy was found in all malignant tumors, we were interested to find out whether these features could also be observed in premalignant somatic lesions. Thus, four available pairs of sufficiently large tumor cohorts were compared: Astrocytoma versus glioblastoma, colon adenoma versus adenocarcinoma, renal adenoma and oncocytoma versus renal carcinoma, and lipoma versus liposarcoma—and salivary gland adenoma was added as the fifth cohort. The results are presented in Table 1 (16–20) and Figure 5. 

### 3.4. Benign Tumors: Study of the Doubled X-Chromosome Karyotypes and Near-Triploidy

In colon adenoma and adenocarcinoma, the proportion of #X-disomic karyotypes with near-triploidy was rather high; however, triploidy in adenoma was lower than in colon adenocarcinoma (11% vs. 16%, respectively). All triploid colon adenoma karyotypes except one were XXY, while the remaining one was XX,-Y (Table 1, Figure 5). In astrocytoma, the total percentage of #X-disomy was more than two times lower compared to glioblastoma, and near-triploidy was also significantly lower (6.78% to glioblastoma’s 10.23%), while liposarcoma surpassed lipoma 4-fold in triploidy, and more than 3-fold in #X-disomy proportions (although both were in the lower range of the two values). The renal adenoma and oncocytoma cohort was more than 4-fold poorer with #X-disomic karyotypes than its malignant counterpart (2.08% vs. 8.49%) and did not show near-triploidy. Salivary gland adenoma also did not display near-triploidy, and #X-disomy was almost absent as well (0.76%).

### 3.5. Principal Component Analysis (PCA)

PCA was applied for the exploration of the X-chromosome disomy (#X-disomy) pattern in order to demonstrate the non-randomness of different #X-disomy categories distribution in near-triploidy across different tumor types. The initial four-dimension space having four different categories of #X-disomy as axes, namely #X-disomy (all types)_62–76 (chromosomes), #X-disomy (all types)_58–80, XXY+XX,-Y_62–76, and #X-disomy (all types)_66–72 and 15 malignant tumor types as statistical units, was submitted to PCA (Table 2).

**Figure 6:**
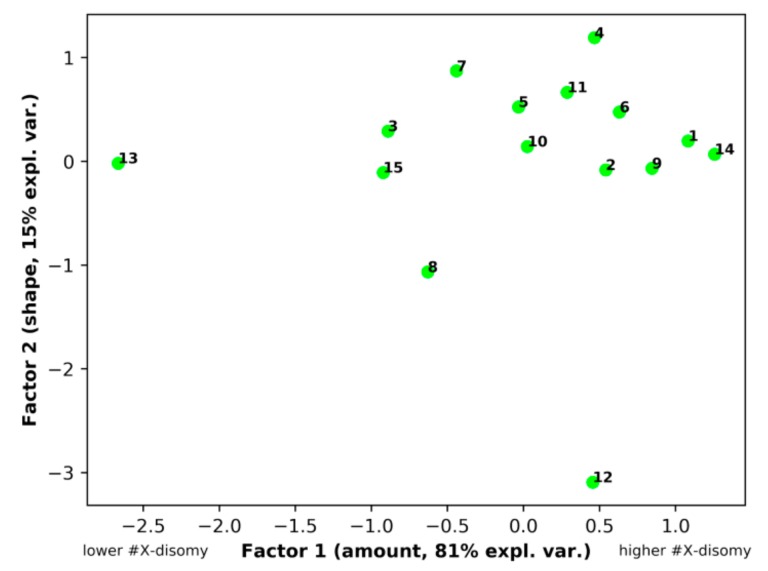
PCA results of the X-chromosome disomy distribution in the triploidy space for 15 malignant tumor types, designated as numbered in Table 1.

As evident from Table 2, the analysis ended up as a two-component solution, cumulatively explaining 96% of the total variance. The #X-disomy distribution highlighted a striking correlation structure, with a major “size” [45], first principal component (Factor1), and a minor “shape” component. The presence of very high and positive loadings (the Pearson correlation coefficient between the original variables and components) for Factor 1 corresponds to the fact that all the categories contribute “along the same direction" to the Factor 1 scores, and thus it is an integrated score of the “amount of #X-disomy". The second “shape” component (Factor 2) explains 15% of the total variance, and this value mainly stems from the unique mesothelioma pattern (see Figure 6). It is worth noting that principal components are orthogonal to each other by construction, and therefore, “size" and “shape" are two independent latent concepts [47]. PCA thus showed an extremely ordered #X-disomy distribution in the near-triploid space of all tumor types. The presence of such a strong correlation structure is indicative of the non-random character of #X-disomy distribution in triploidy. As a matter of fact, a maximum likelihood approach to factor extraction [46] highlighted a very high statistical significance (Bartlett-corrected chi-square = 87.01, *p* < 0.0001) against the null hypothesis of no common factor. 

Commenting on the space distribution of #X-disomy for different tumor types, it should be noted that 12 of 14 somatic tumor types form a common “shape” cluster with the germ tumor seminoma underlying a common biological phenomenon. The peculiarity of mesothelioma (Nr 12) may be due to its (still unknown) highly plastic progenitors switching between different phenotypes depending on the local microenvironment [48]. Thus, the PCA results showed an extremely ordered X-chromosome disomy distribution in the near-triploid space of all tumor types. The presence of such a strong correlation structure is indicative of the non-random character of #X-disomy distribution in triploidy. The emergence of a greatly major “size" component points toward a largely invariant pattern of disomy distribution among tumor types that mainly differ by the amount of #X-disomy, keeping the relative frequency of various tumor types largely constant.

## 4. Discussion

In this study, we hypothesized that tumor near-triploidy, which is associated with chemoresistance, may originate primarily from the rearrangement of whole-genome complements. Therefore, we attempted to differentiate it from aneuploidy resulting from chromosomal aberrations. As a method, we chose to analyze the sets of sex chromosomes in relation to modal chromosome numbers in male tumor karyotypes. Through all analyzed material representing 20 types of 2928 epithelial and mesenchymal, somatic and germ, malignant and benign male tumor karyotypes, we found that near-triploid karyotypes were characterized by X-chromosome disomy. 

The simplest explanation for #X-disomic chromosomes would be their origin from the mis-segregation of sister chromatids in aberrant mitosis. The random mis-segregation of individual chromosomes is a well-known mitotic aberration in breakage–fusion–bridge cycles. Tumor aneuploidy concerning the re-arrangements of whole chromosomes and near-triploidy, in particular, has been hitherto explained mostly from the position of the random aberrations in mitotic cycles [2]. However, Pearson correlations carried out in our study established that a kind of aneuploidy represented by #X-disomy (with the dominating XXY karyotypes) showed a very high correlation (*r* ~ 0.9, *p* < 0.001) with triploidy, while PCA resulted in an extremely ordered X- chromosome disomy distribution in the near-triploid space of all tumor types, clearly indicating a departure from randomness.

In addition, #X-disomic tumors were clearly repulsing from the near-diploid chromosome range with a strong negative correlation, and thus collectively depart from the null hypothesis of the random origin of X chromosome mis-segregation.

It is also worth noting, in addition, that the dominating set of #X-disomy in male cells was XXY, while the XYY sets, which at random would exhibit a 50% possibility, were practically non-existent in the dataset (with only one among 2928 analyzed male tumor karyotypes being near-triploid XYY). This indicates that #X-disomy and near-triploidy of male tumor karyotypes are two facets of the same phenomenon, rather than stemming from random aberrant chromosome mis-segregation in a mitotic cycle. 

We also found that somatic tumor types and seminoma are not only highly correlated together with #X-disomy related to near-triploidy by the Pearson coefficient and *p*-value, but also constituted a common “shape” cluster in PCA space. So, the identified novel phenomenon of the persuasive link between #X-disomy and near-triploidy dominated by the “feminized” XXY karyotype may not be of mitotic origin. Rather, it may possess meiotic features programmatically directed towards the oogenic pathway.

Although the above analysis points towards the concerted mis-segregation of the chromosome complement of the maternal genome, which would be expected in a process similar to oocyte development, the direct data confirming this process have not yet been elaborated. However, the study of Ozery-Flato et al. [49] on the same Mitelman database including 15,000 karyotypes of 62 tumor cohorts, among them 18 solid (some the same as we have explored), analyzing all aberrations identifiable by cytogenetic techniques, revealed the strongest association among mainly whole chromosome gains and losses, with the gains prevailing. This regularity was also confirmed by comparative genome hybridization analysis. The data of these researchers are supportive for our interpretation of XXY male tumor triploid karyotypes as resulting from the whole genome complements rearrangements of meiotic (for somatic tumors, pseudo-meiotic) origin. 

How may this process proceed? XXYY sets could possibly serve as a starting point. A configuration of XXYY sex chromosomes was found to be present in each malignant tumor cohort of our tumor sets (except for gastric carcinoma) and was particularly high in seminoma (10.3%). Although designated as triploid XXY,+Y (see the last column in Table 1), it can derive either from spermatocytes I or from the G2/mitotic slippage fraction of XXYY of any tumor. #X-disomic karyotypes did not display a statistically significant correlation with tetraploidy (Figure 2F). In other words, the absence of this correlation does not prove—but also does not deny—that the formation of XXY triploid karyotypes for male tumors can start from the G2/mitotic slippage (XXYY) phase as a meiosis-like process. In addition, our study of breast cancers showed that triploid stemlines contain, in addition, four times more ≥4C cells than the populations with a near diploid stem line [29].

Molecularly, and particularly in the case of reprogramming, this mito-meiotic trigger is feasible because the G2 mitotic recombination checkpoint is identical to the recombination checkpoint of the meiotic prophase and evolutionary derived from it [28,50]. The reprogramming shift may be favored by cell senescence and associated DNA damage [51], chromosome instability (CIN) itself [3], and also by the hyper-activation of the RAS-RAF/MOS-MEK-MAPK pathway, where the up-regulated RAS can induce either senescence or also be substitutive for MOS in oocyte maturation and active in fertilization [51,52]. In accordance with this, human somatic tumors ectopically express meiotic genes and proteins, and also enhance their synthesis after genotoxic challenge [53,54,55,56,57,58,59], while primordial male germ cells and their immediate progeny are able to undergo oogenesis in adverse conditions [60].

**Figure 7:**
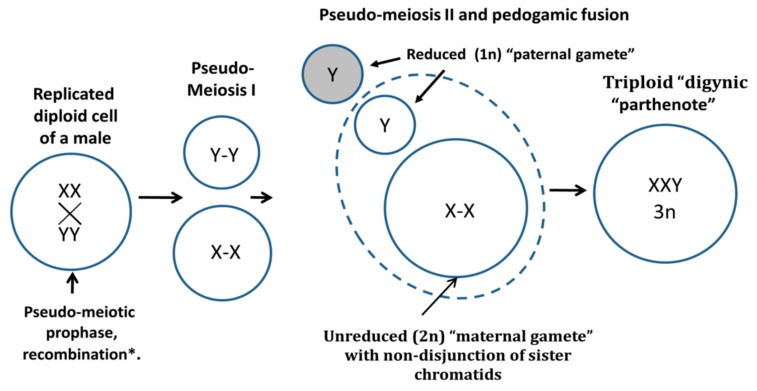
Schematic of the digyny-like formation of XXY triploid karyotypes in somatic male tumors. The reprogrammed male tumor cell triggers the aberrant molecular pathway of the pseudo-meiotic prophase from the G2-phase, undergoes recombination between cohered sisters and possibly also homologues, undergoes pseudo-meiosis I segregating maternal and paternal progenies with cohered sister chromatids, and triggers the reduction to haploidy of the “paternal gamete” in the pseudo-meiosis II and its pedogamic fusion with the unreduced diploid “maternal gamete”, resulting in triploid “digynic parthenote”.

If so, a process similar to sexual digyny (the aberrant fusion of an unreduced maternal diploid gamete possessing the non-disjunct sister chromatids with a paternal haploid gamete), occurring here in a parasexual, pedogamic manner, could explain the persuasive formation of XXY triploid karyotypes in a proportion of male tumors. It is schematically presented on Figure 7 as starting with a glide into a meiotic prophase-like state from the mitotic G2 recombination checkpoint. The trigger between this survival pathway and death in mitotic catastrophe may depend on the actual concentration of the MOS-dependent undegraded cyclin B1 [28,51,61], while chromothripsis with its micronucleation may be part of this decision threshold [62,63].

The digyny-like process can use meiotic recombination for the effective repair of DNA damage and gene conversion, while the third genome can compensate for recessive lethal mutations (and also moderate the genome imbalance created by the inevitably joined CIN). Thus, this variant of the whole genome aberrations in triploid tumors can favor both their perpetuation and chemoresistance. More information on the evolutionary significance of digyny for adaptation to catastrophic environments and the alternation of the digyny cycle with a mitotic cycle in triploid tumors can be found in [64]. 

Our findings lead us to question if these revelations have any potentially predictive clinical significance. To this end, it is interesting to compare somatic tumors and seminoma, on one side, and colon adenoma and carcinoma, on the other side. Table 1 shows that seminoma has the highest proportion of XXY karyotypes (47%) and also a fraction of XX,-Y - likely due to the secondary loss of the Y-chromosome (together 56%); however, it possesses a relatively small fraction (5%) of less stable #X-disomic karyotypes (XY,+X)+(X,-Y,+X). On the contrary, somatic tumors display a higher proportion of more defective karyotypes—which, however, still maintain #X-disomy—and they still negatively correlate with near-diploidy. This points towards a primary origin of XXY triploidy and a secondary origin of unstable #X-disomic karyotypes derived from it, and also highlights the fact that somatic tumors have higher secondary CIN (involving the loss of single chromosomes, #Y and autosomes) than seminoma. In accordance with our suggestion that seminoma is less subjected to secondary CIN alterations, it was found that, except for a few driver mutations, seminoma has far less secondary passenger mutations in comparison with somatic cancers [65]. In the case of colon cancer, where the tumor suppressor adenomatous polyposis coli (*APC*) function loss occurs in early adenoma, the acquisition of a driver mutation *KRAS* in late adenoma, while the exaggerated chromosome and microsatellite instability further develops with cancer progression and loss of *TP53* function [66], the secondary stochastic CIN process degrading the triploidy partly overlaps and likely masks the germinative initiation by triploidy. Therefore, the colon adenoma is enriched with XXY triploidy, while adenocarcinoma accumulates, in addition, the near-diploid #X-disomic karyotypes. Interestingly, and in accordance with this interpretation, Giaretti and colleagues [67] found that the incidence of mutations of the *KRAS2* and *TP53* genes was lowest among the DNA near-triploid and highest among the near-diploid cases of sporadic colorectal adenocarcinoma. Deeper investigation into the predictive potential of our findings is needed.

## 5. Conclusions

The analysis of the karyotypes of 15 male malignant tumor types, germ and somatic, from the Mitelman database revealed a very high correlation between Xchromosome disomy (predominantly represented by XXY karyotypes) with triploidy, a negative correlation with near-diploidy, and no correlation with near-tetraploid modal chromosome numbers. In addition, principal component analysis revealed the strongly non-random nature of the #X-disomy and triploidy association and clustering of the germ tumor seminoma with somatic tumors in the PCA space. Collectively, this suggests that the disomy of the X-chromosome and triploidy in the typically XXY karyotypes in male tumors represent two facets of the same biological phenomenon: The rearrangement of the whole genome complements. A hypothesis of a digyny-like process (the aberrant fusion of two maternal genomes with one paternal genome) exploiting the elements of meiosis in reprogrammed tumor cells has been proposed. The analysis of partly defective XXY triploid karyotypes still maintaining #X-disomy allows us to suggest that chromosome instability may be largely secondary to the whole genome complement rearrangements. The potential for clinical predictions based on the comparison of colon adenoma and carcinoma and of seminoma with somatic malignant tumors is preliminarily discussed. 

## Figures and Tables

**Table 1:** The analyzed male tumor types, the number of karyotypes per cohort, the percentage share of near-triploidy (in the range of 62–76 chromosomes), and the percentage share of sex chromosome configurations containing a disomic X-chromosome. The karyotype XXY in most cases indicates near-triploidy with the three sex chromosome complement XXY. XX,-Y largely means a near-triploid karyotype from a male with the loss of the Y chromosome, where "-Y" indicates the third haploid chromosome set. Sex chromosomes XY,+X indicate a near-diploid male karyotype with the (not inherited) acquisition of the extra sex chromosome X. The diploid (or near-diploid) male karyotype X,-Y,+X means the (acquired) loss of sex chromosome Y and gain of sex chromosome X, while karyotype XXY,+Y is near-triploid by chromosome number, with a gain of chromosome Y.

Nº	Malignant Tumor Type	Number of Karyotypes	% of Near- Triploidy (62–76)	XXY %	XX,-Y %	(XY,+X)+(X,-Y,+X) %	XXY,+Y %
1	Seminoma	78	42.31	47.44	3.85	5.13	10.26
2	Osteosarcoma	61	27.87	24.59	3.28	0.00	6.56
3	Lung carcinoma	237	27.00	8.02	9.70	2.53	2.53
4	Gastric carcinoma	74	20.27	10.81	5.41	6.76	1.35
5	Head and neck squamous cell carcinoma	191	16.75	5.76	8.90	0.52	1.57
6	Colon adenocarcinoma	98	16.33	12.24	6.12	10.20	6.12
7	Transitional cell carcinoma	104	13.46	4.81	3.85	1.92	1.92
8	Chondrosarcoma	85	11.76	4.71	3.53	3.53	0.00
9	Malignant melanoma	134	10.45	5.22	3.73	2.24	2.24
10	Glioblastoma	215	10.23	7.44	1.40	0.00	1.40
11	Renal carcinoma	577	7.11	3.81	4.68	1.04	1.21
12	Mesothelioma	72	6.94	5.56	2.78	0.00	2.78
13	Rhabdomyosarcoma	92	6.52	3.26	1.09	3.26	0.00
14	Ewing sarcoma	228	3.51	3.95	0.00	3.51	0.88
15	Liposarcoma	147	3.40	1.36	1.36	0.00	0.00
	**Benign tumor type**						
16	Colon adenoma	62	11.29	11.29	4.84	0.00	0.00
17	Astrocytoma	59	6.78	1.69	1.69	1.69	1.69
18	Lipoma	235	0.85	0.85	0.00	0.00	0.43
19	Renal adenoma and oncocytoma	48	0.00	0.00	2.08	0.00	0.00
20	Salivary gland adenoma	131	0.00	0.00	0.76	0.00	0.00

**Table 2:** Principal component analysis (PCA) loading pattern.

Original Variables	Factor1	Factor2
#X disomy_62-76	0.96650	−0.23119
#X disomy_58-80	0.94894	−0.05406
XXY+XX,-Y_62-76	0.95938	−0.23271
#X disomy_66-72	0.69858	0.71287
% of Explained Variance	81.1	15.5
